# Cell Signaling in Model Plants

**DOI:** 10.3390/ijms21176062

**Published:** 2020-08-23

**Authors:** Jen-Tsung Chen, Parviz Heidari

**Affiliations:** 1 Department of Life Sciences, National University of Kaohsiung, Kaohsiung 811, Taiwan; 2 Department of Agronomy and Plant Breeding, Faculty of Agriculture, Shahrood University of Technology, Shahrood 3619995161, Iran; heidarip@shahroodut.ac.ir

Plants as sessile organisms are not able to move and must cope with adverse environmental conditions and stresses such as extreme temperatures, drought, high soil salinity, oxidative stress, pathogen attack, and so on. To respond in an appropriate manner to a specific environmental stimulus, plants possess signal transduction pathways, which are complex networks of interactions involving signal elements transmitting through the plant cell. In fact, cell signaling affects virtually every aspect of plant cell structure and function. For building elegant, complicated, and interconnected regulating networks, a huge number of components are involved, including receptors, secondary messengers, protein kinases, transcription factors, reactive oxygen species (ROS), and plant hormones that regulate or stimulate other components. Therefore, to unveil a global picture of plant cell signaling networks and underlying master regulators and machinery remains a challenge for researchers. For enriching our understanding of plant cell signaling with the assistance of modern molecular tools, this Special Issue “Cell Signaling in Model Plants” collects recent innovative original research and reviews, with an emphasis on the studies using model plants and crops. A total of 14 publications were published, which can be divided into five subtopics, as described below.

## 1. Arabidopsis Thaliana Transmembrane Receptor-Like Kinases (RLKs)

Transmembrane receptor-like kinases (RLKs) are conserved protein kinases that play critical roles in transducing the signal from the outside to the inside of plant cells, particularly to the nucleus. Jose et al. refined the recent advances of transmembrane RLKs in *A. thaliana* to unveil how stress responses as well as plant development are regulated by signaling pathways related to different groups of RLKs [1].

## 2. Functional Analysis of Signaling Components 

### 2.1. Flowering Locus C (FLC) Homologs

To reveal the underlying mechanisms controlling the annual flowering of fruit trees, Kagaya et al. analyzed the function of *FLOWERING LOCUS C* (*FLC*) homologs in apple [2]. They declared that homologs of *FLC* might be involved in flowering and associated with juvenility.

### 2.2. Della/Gai Functions 

Gibberellin (GA) signaling plays a vital role in regulating plant growth and development. Wang et al. investigated the function of GAI-1, one of the proteins with the DELLA amino acid motif (aspartic acid–glutamic acid–leucine–leucine–alanine), as a negative regulator involved in GA signaling, using the *gai-1* mutant line of *A. thaliana* [3]. They indicated that the *gai-1* mutant line is more tolerant of drought than the wild type. They also found a strong interaction between GAI proteins and ABA-responsive element (ABRE)-binding transcription factors. Jung et al. characterized *SLR1*, encoding the DELLA protein in rice, using CRISPR/Cas-9 genome editing [4]. They stated that the expression of GA_20_OX_2_ (gibberellin oxidase) and GA_3_OX_2_, as GA-related genes, was upregulated in the edited mutant lines. Finally, they indicated that in the created mutant lines, *slr1-d7* and *slr1-d8*, cell elongation is limited. 

### 2.3. Dwarf Gene Mini Plant 1 (MNP1)

Dwarf phenotypes are widely used in crop breeding to increase resistance and yield. Guo et al. defined the function of *MNP1* in *Medicago truncatula* as a model legume plant [5]. They concluded that in the *mnp1* mutant line, the cell number of internodes and cell length are reduced. They also found that MNP1 is committed in GA biosynthesis.

### 2.4. MiPEP165a

MicroRNAs (miRNAs) play a significant role in the regulation of gene expression. Ormancey et al. investigated the role of a miRNA-encoded peptide, miPEP165a, in *A. thaliana* [6]. The authors found that passive diffusion followed by an endocytosis process are two functions of entry of miPEP165a.

## 3. Cell Signaling in Response to Biotic Stresses

Ethylene is a gaseous phytohormone that is involved in response to biotic and abiotic stresses. Zhang et al. studied the effect of ethylene signaling on resistance to some aphids in *Medicago truncatula* using an ethylene-insensitive mutant called sickle mutant [7]. Their results revealed that the sickle mutant can cause a moderate resistance to some aphids from the independent pathway of R-genes such as *AKR* (*Acyrthosiphon kondoi* resistance), *APR* (*Acyrthosiphon pisum* resistance), and *TTR* (*Therioaphis trifolii* resistance).

*Botrytis cinerea* is a necrotrophic fungus causing grey mold disease in many plant species. Maqsood et al. examined the effect of iprodione, as a fungicide, on the molecular mechanisms of *B. cinerea* resistant mutant [8]. By analyzing the whole transcriptome sequencing, they pronounced that genes involved in metabolism, production of detoxification enzymes, mitogen-activated protein kinases (MAPK) signaling, transporter function, catalytic activity, and drug efflux are linked with resistance to iprodione.

## 4. Cell Signaling Related to Plant Acclimation

### 4.1. Brassinosteroid Signaling

Brassinosteroids (BRs) as steroid hormones play critical roles in regulating the plant growth and development stages. Mao and Li reviewed the regulatory mechanisms of brassinosteroid-insensitive 1 (BRI1), BRI1-associated receptor kinase (BAK1), and brassinosteroid-insensitive 2 (BIN2), as three key kinases, involved in the BR signaling cascade [9]. They stated that BIN2, as an interface kinase, plays an important role in BR signal transduction from receptor kinase, BRI1/BAK1, to nucleus through two transcription factors, BRI1-EMS-supressor 1 (BES1), and brassinazole-resistant 1 protein (BZR1). 

BRs have many interactions with other phytohormones that regulate the downstream pathways related to plant growth or response to stresses. Bulgakov and Avramenko reviewed the links between BR and abscisic acid (ABA) signaling that affect the stress-acclimation processes [10]. They proposed three interconnected mechanisms that in the first mechanism, BIN2, as a kinase of BR signaling, is responsible for interaction with ABA signaling. 

### 4.2. Cyclic AMP Signaling

The cyclic nucleotide, cAMP (3′,5′-cyclic adenosine monophosphate) as a signaling molecule, is involved in molecular processes linked to response to environmental stresses. Blanco et al. reviewed the current knowledge of cAMP signaling [11]. They indicated that the main signaling mechanism of this cyclic nucleotide is exchange of cAMP into Ca^2+^ signals through cyclic nucleotides-gated channels.

### 4.3. Hydrogen Sulfide

Hydrogen Sulfide (H_2_S) is a toxic gaseous molecule. Recent studies revealed that H_2_S plays a positive role in regulating plant growth. Xuan et al. considered the H_2_S roles in cellular processes and also indicated all possible crosstalk between H_2_S and phytohormones [12]. Interestingly, the authors suggested that H_2_S may affect the protein activities and subcellular localization by contributing to post-translational modification. 

## 5. Effects of Selenium and Sedimentary Calcite-Processed Particles on Cell Signaling

Selenium (Se) is identified as a beneficial element that can be included in improving the plant’s resistance facing adverse environmental conditions. Kamran et al. reviewed the protectant roles of Se in response to soil salinity [13]. They indicated that Se can improve salinity tolerance by decreasing Na^+^ ion accumulation through the expression induction of the Na^+^/H^+^ antiport and increasing the antioxidants.

Tran et al. investigated the early cellular responses of bright yellow2 tobacco cultured cells under the application of calcite processed particles (CaPPs) [14]. Their results revealed that CaPPs act such as nanoparticles and induce various signaling pathways. CaPPs firstly induced ROS and then, increased the cytosolic Ca^2+^ and activation of anion channels.

## 6. Conclusions and Perspectives

This Special Issue provides new and in-depth insights into molecular aspects of plant cell signaling in response to biotic, such as aphid- and grey mold disease-resistance, and abiotic stresses, such as soil salinity and drought stress, and additionally, functional analysis on signaling components involved in flowering, juvenility, GA signaling, and biosynthesis, and miRNA-regulated gene expression. Furthermore, plant acclimation was reported, with emphasis on mechanistic insights into the roles of brassinosteroids, cyclic AMP, and hydrogen sulfide, and the recent advances of transmembrane receptor-like kinases were refined. Clearly, plant cell signaling is an intensive topic and whether it is now or in the future, the emerging technology in functional analysis such as genome editing technologies, high-throughput technologies, integrative multiple-omics as well as bioinformatics can assist researchers to reveal novel aspects of the regulatory mechanisms of plant growth and development, and acclimation to environmental and biotic stresses. The achievement of such research will be useful in improving crop stress tolerances to increase agricultural productivity and sustainability for the food supply of the world.
